# Factors associated with complications related to the use of peripherally inserted central venous catheter in hospitalized children: a case-control study, Sorocaba, Brazil, 2018-2022

**DOI:** 10.1590/S2237-96222025v34e20240134.en

**Published:** 2025-09-08

**Authors:** Mayara Amancio de Souza, Luciane Cruz Lopes, Marcus Tolentino Silva

**Affiliations:** 1 Universidade de Sorocaba, Programa de Pós-Graduação em Ciências Farmacêuticas, Sorocaba, SP, Brasil; 2 Universidade Federal de Brasília, Departamento de Saúde Coletiva, Brasília, DF, Brasil

**Keywords:** Catheter Obstruction, Central Venous Catheters, Peripheral Catheterization, Pediatrics, Case and Control Studies, Obstrucción del Catéter, Catéteres Venosos Centrales, Cateterismo Periférico, Pediatría, Estudios de Casos y Controles

## Abstract

**Objective:**

To evaluate factors associated with complications related to the use of peripherally inserted central venous catheters in children undergoing surgical procedures.

**Methods:**

This was a case-control study, conducted in a public hospital in Sorocaba, with an emphasis on surgical procedures, between 2018 and 2022. Electronic medical records were used as a source of information. Odds ratio (OR) and 95% confidence intervals (95%CI) were calculated.

**Results:**

The study included 109 children aged up to 2 years 11 months and 29 days of age, of which 62 were cases and 47 were controls. The frequency of complications related to the use of peripherally inserted central venous catheters was 56.9% (95%CI 47.3; 66.0), and the most frequent complications were obstruction (30.3%) (95%CI 22.3; 39.7), accidental removal (10.1%) and hyperemia (9.2%). There was a greater chance of complications when the child remained in the neonatal pediatric intensive care unit (OR 5.45; 95%CI 1.20; 24.8) and when maintaining intravenous hydration (OR 3.71; 95%CI 1.02; 13.52). Female children (OR 2.93; 95%CI 1.24; 6.91), using total parenteral nutrition (OR 2.97; 95%CI 1.11; 7.94) and with Down syndrome (OR 19.19; 95%CI 2.23; ∞) had a higher chance of obstruction.

**Conclusion:**

Children in intensive care and receiving intravenous hydration had a higher chance of complications. Understanding the factors associated with complications benefits patients, healthcare institutions and the Brazilian Unified Health System (SUS).

## Resumen

**Objetivo:**

Verificar los factores asociados a las complicaciones relacionadas con el uso de catéteres venosos centrales de inserción periférica en niños sometidos a procedimientos quirúrgicos.

**Métodos:**

Se trata de un estudio de casos y controles, realizado en un hospital público de Sorocaba, con énfasis en procedimientos quirúrgicos, entre 2018 y 2022. Se utilizó la historia clínica electrónica como fuente de información. Se calcularon los odds ratio (OR) y los intervalos de confianza del 95% (IC95%).

**Resultados:**

Se incluyeron en el estudio 109 niños de hasta 2 años, 11 meses y 29 días, de los cuales 62 fueron casos y 47 controles. La frecuencia de complicaciones relacionadas con el uso de catéteres venosos centrales de inserción periférica fue de 56,9% (IC95% 47,3; 66,0), y las más frecuentes fueron obstrucción (30,3%) (IC95% 22,3; 39,7), retiro accidental (10,1%) e hiperemia (9,2%). Hubo mayor probabilidad de complicaciones cuando el niño permaneció en la unidad de cuidados intensivos pediátricos neonatales (OR 5,45; IC95% 1,20; 24,8) y cuando mantuvo hidratación intravenosa (OR 3,71; IC95% 1,02; 13,52). Las niñas (OR 2,93; IC95% 1,24; 6,91), que utilizaban nutrición parenteral total (OR 2,97; IC95% 1,11; 7,94) y con síndrome de Down (OR 19,19; IC95% 2,23; ∞) tuvieron mayor probabilidad de obstrucción.

**Conclusión:**

Los niños en cuidados intensivos y recibiendo hidratación intravenosa tuvieron mayor probabilidad de presentar complicaciones. Comprender los factores asociados a las complicaciones beneficia a los pacientes, a las instituciones de salud y al Sistema Único de Salud.

**Ethical aspects**

This research respected ethical principles, having obtained the following approval data::

Research Ethics Committee: Universidade de Sorocaba

Opinion number: 5.973.039

Approval date: 29/3/2023

Certificate of Submission for Ethical Appraisal: 66973123.6.0000.5500

Informed Consent Form: Exempted.

## Introduction

In pediatric patients, the most common invasive procedure during hospitalization is the insertion of intravenous catheters (1). Most children, especially those undergoing surgery, require vascular access to administer medications and perform tests. These devices are essential for treatments such as hydration, infusion of antibiotics, vesicant drugs, blood components, total parenteral nutrition, and medications for hydroelectrolytic balance (1,2). In newborns and infants, ensuring intravenous access is still a challenge due to difficulties in puncture and maintenance, as the venous network has a smaller caliber and less visibility during this period (3). 

Peripherally inserted central venous catheter (PICC), is a safe option and is increasingly used in pediatric patients, with advantages such as prolonged intravenous therapy, preservation of the venous network, reduction of pain and stress and administration of hypertonic solutions. Its advantages, compared to other central catheters, are its lower cost, lower risk of infection, lower incidence of pneumothorax, and greater patient mobility (3,4). The peripherally inserted central venous catheter can be inserted at the bedside by trained nurses (4).

Despite the benefits, PICC is not without complications. Among pediatric patients, the most common complications include thrombosis, obstruction, intravenous infiltration, bloodstream infection, and potentially fatal complications such as pleural effusion, pericardial tamponade, and pericardial effusion (5).

In Brazil, few studies have evaluated the use of PICC in the Unified Health System in pediatric surgical patients. Through the case-control study, it is possible to understand the factors associated with the occurrence of complications in children in this age group. Understanding the associated factors can favor actions aimed at reducing complications in healthcare institutions. These initiatives can promote benefits to patients and the Unified Health System, by optimizing resources and improving the quality of care provided. The objective of this research is to evaluate the factors associated with the occurrence of complications related to the use of PICC in children undergoing surgical procedures.

## Methods

### Study design

This was a case-control study. All children underwent PICC insertion. The case group consisted of patients who had some type of complication. A complicaiton was considered the primary outcome of this study: non-elective removal of the device, such as accidental removal, death, hyperemia, obstruction, deep vein thrombosis and infection during the hospitalization period. 

The control group was defined as: patients who used PICC in the same period and had no complications; hospital discharges;quality deviations, which were identified when the PICC did not meet the quality parameters; failures in connectors adaptation; in addition to those that required counter-referral with the PICC inserted, but without monitoring the removal of the device. 

### Context

The research was conducted at the Hospital Regional de Sorocaba “Dr. Adib Domingos Jatene,” a public hospital located in Sorocaba, São Paulo, after approval by the ethics committees of the institution and Universidade de Sorocaba. Managed by the Associação Paulista para o Desenvolvimento da Medicina (São Paulo Association for the Development of Medicine), in partnership with the São Paulo State Department of Health, the hospital is a tertiary reference in surgical specialties such as orthopedics and traumatology, circulatory system surgery, general surgery and trauma, neurosurgery, pediatric surgery, and clinical medicine with an emphasis on acute myocardial infarction and cerebrovascular accident. 

The institution has 144 beds, of which 26 are pediatric. Of these, 16 are for the surgical pediatric ward, and 10 are for the neonatal pediatric surgical intensive care unit, with an emphasis on congenital heart disease and pediatric surgeries. In May 2024, the institution obtained recertification of Accreditation with Excellence, level 3, by the National Accreditation Organization. Accreditation with Excellence recertification is the reassessment conducted by an accrediting entity to confirm that a healthcare institution maintains high standards of quality and safety in patient care (6).

The purchase of PICC and other materials in the hospital is conducted in a consolidated manner. After the orders are consolidated, announcements and publications are made for all suppliers registered on the Electronic Portal. The bid selection criterion is the lowest price, as long as it meets the technical specification. The product is not purchased in cases of brand restriction by the National Health Surveillance Agency or ban by the institution’s Supplier Qualification Technical Committee. This acquisition organization prevents the standardization of PICC regarding the quality of materials (silicone or polyurethane), the number of lumens, measurements in French (Fr) and the presence of a microintroducer for direct or ultrasound-guided punctures.

### Participants

The research participants were children who underwent surgical procedures and used PICC at the institution between 2018 and 2022. The sample included 109 children, subdivided between: 0 and 28 days; 29 days to 1 year; and up to 2 years, 11 months, and 29 days of age. Children over 3 years old, records of unsuccessful PICC insertions and cases in which despite the request for PICC insertion the central venous catheter was inserted were excluded.

### Variables

For sample characterization, frequencies and measures of central tendency and the dispersion of some variables were calculated. The year of PICC insertion was considered during the 2019 coronavirus pandemic. Demographic variations included sex (male, female) and age (in months). Clinical variables included the presence of comorbidities (hypertension, dyslipidemia, heart disease, immunosuppression, kidney disease, lung disease and Down syndrome), types of surgeries performed (general, cardiac, neurological, urology), infusion therapy modalities (parenteral nutrition, medications) and PICC insertion sites (upper limbs, lower limbs, jugular, femoral, axillary, cephalic veins). Regarding the variables related to the PICC, the number of insertion attempts, the catheter’s indwelling time, type of puncture (direct, ultrasound-guided), caliber used, reason for PICC removal and the occurrence of complications were analyzed.

### Data sources and measurement

Data was collected in 2023. As a source of information, the electronic medical record and the hospital management operating system were used during the hospitalization period between 2018 and 2022.

To extract the data, a questionnaire was prepared by the author using the KoBoToolbox tool. Mobile electronic devices were used to record the information. During the information collection, carried out by the author of the research, the data was constantly verified by checking the information uploaded to the KoBoToolbox platform, to identify and correct any inconsistencies, such as failure to load the collected data. 

### Bias control

To reduce information bias in relation to data collection, the dispensing of the PICC to the patient was identified through the institution’s cost center. Based on this information, a systematic check of the data in the electronic medical record was conducted, starting from the date the PICC was dispensed, which made it possible to correct any inconsistencies.

To mitigate the confounding bias resulting from the severity of the patients, since the hospital profile serves those with a higher risk of complications, multivariate analyses were performed considering age as a surrogate indicator of prognosis.

### Study size

Using the electronic medical records, all children with PICCs in the institution were searched for, so the sample size calculation was not performed. Considering the number of cases and controls available, power was calculated as a 95% confidence level.

### Statistical methods

Categorical variables were analyzed using chi-square test, or Fisher’s exact test when appropriate. P-values less than 0.05 were considered statistically significant.

To investigate the measures of association between the most frequent complications, univariate and multivariate logistic regression models were used. The results were presented as odds ratio (OR) adjusted by age group and sex, with 95% confidence intervals (95%CI). When necessary, the exact logistic regression model was used to better deal with excess positive results for the outcome. The same routine was applied with the most frequent complication in the study. All calculations were performed in STATA 14.2.

## Results

**Figure 1:**
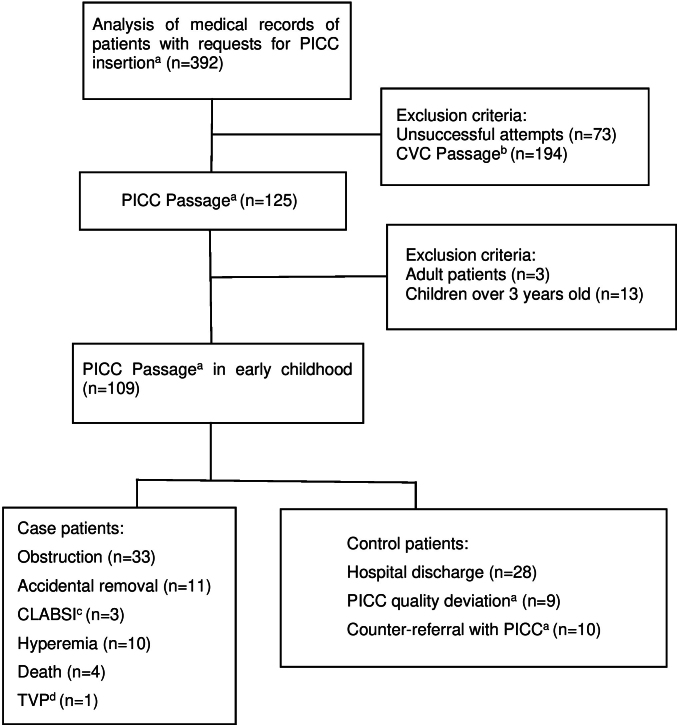
Flow of inclusion and selection of patients in the study. Sorocaba, 2018-2022

A total of 392 medical records with requests for PICC insertion were analyzed and 125 insertions were performed (Figure 1). Of these, 109 children were included in the research, of which 62 cases and 47 controls were identified. The power of the study was estimated at 89.0% considering hospitalization in a pediatric intensive care unit as the exposure factor.

The sample of this research consisted of 109 individuals who used PICC (Table 1). The pandemic period did not influence the results of the study. The highest frequency of PICC insertion occurred in children aged 29 days to 1 year. Males represented most insertions compared to females. The highest frequency regarding the indwelling time of the PICC was between 0 and 10 days. The most frequent puncture site chosen was the upper limbs and, in relation to the laterality of the puncture position, on the right side. The triple lumen catheter was included with the double lumen catheter due to the occurrence of only one observation.

**Table 1:** Demographic, clinical, and intravenous therapy characterization of children in the case and control groups. Sorocaba, 2019-2022 (n=109)

	Sample	Cases	Control	
Variable	n (%)	n=62	n=47	p-value^a^
Year				0.661
2019	13 (11.9)	9	4	
2020	39 (35.8)	23	16	
2021	42 (38.5)	23	19	
2022	15 (13.80)	7	8	
**Age range**				0.528
0-28 days	32 (29.4)	16	16	
29 days to 1 year	54 (49.5)	31	23	
Up to 2 years	23 (21.1)	15	8	
Gender				0.072
Male	66 (60.6)	33	33	
Female	43 (39.5)	29	14	
**Days with PICC^b^**				0.366
0-10	46 (45.1)	31	15	
11-20	32 (31.4)	17	15	
21 or more	24 (23.5)	13	11	
**Puncture site**				0.595^c^
Upper limb	56 (51.4)	31	25	
Cephalic	9 (8.3)	7	2	
Jugular	36 (33.0)	19	17	
Lower limb	8 (7.3)	5	3	
**Puncture position**				0.121
Right	53 (53.0)	33	20	
Left	47 (47.0)	22	25	
**Superior vein**				0.589^c^
Basilic	23 (42.6)	12	11	
Cephalic	8 (14,8)	3	5	
Axillary	8 (14,8)	4	4	
Cubital median	15 (27.8)	10	5	
**PICC caliber^b^**				0.723^c^
1.9 Fr	25 (22.9)	15	10	
2 Fr	73 (67.0)	42	31	
3-4 Fr	11 (10.0)	5	6	
**Lumen number**				0.923
1	33 (30.3)	19	14	
2-3	76 (76.0)	43	33	
**Puncture attempts**				0.707^c^
1	76 (69.7)	45	31	
2	11 (10.0)	6	5	
3	10 (9.2)	4	6	
4-5	12 (11.0)	7	5	
**Place of hospitalization**				0.097^c^
Pediatric ward	10 (9.1)	3	7	
NICU^d^/pediatric	99 (99.8)	59	40	
**Type of surgery**				
General	34 (31.2)	20	14	0.783
Cardiac	67 (61.5)	39	28	0.724
Neurological	18 (16.5)	13	5	0.150
Urological	2 (1.8)	0	2	0.184^c^
Comorbidities				
Hypertension	1 (0.9)	0	1	0.431^c^
Dyslipidemia	2 (1.8)	2	0	0.505^c^
Heart disease	74 (67.9)	43	31	0.707
Immunosuppression	5 (4.6)	3	2	1,000c
Kidney disease	1 (0.9)	1	0	1,000c
Pneumopathy	8 (7.3)	4	4	0.724^c^
Down syndrome	4 (3.7)	4	0	0.132^c^
**Use of inputs**				
Parenteral nutrition	33 (30.3)	23	10	0.075
Antibiotic	99 (99.8)	59	40	0.072
Antifungal	2 (1.8)	1	1	1.000c
Hydration	96 (88.1)	58	38	0.043
Vesicants and irritants^e^	78 (71.6)	48	30	0.119
**Access difficulty**	108 (99.1)	62	46	0.431^c^

^a^Pearson’s chi-square test; ^b^PICC – peripherally inserted central catheter; ^c^Fisher’s exact test; ^d^NICU – neonatal intensive care unit; ^e^Medications considered: aminophylline, amiodarone, sodium bicarbonate, calcium chloride, potassium chloride, sodium chloride, diazepam, dobutamine, dopamine, epinephrine, phenylephrine, phenytoin, calcium gluconate, mannitol, metaraminol, norepinephrine, promethazine, thiopental, and vasopressin.

All punctures were performed directly, without the aid of ultrasound. The basilic vein was the most frequent in upper limb puncture. Regarding the characteristics of the PICC, all were without a valve tip and made of silicone, with the most used gauge being 2 French and a double lumen catheter. The location with the highest frequency of PICC insertion was the neonatal pediatric intensive care unit, and the data demonstrated that most professionals were successful on the first puncture attempt. Heart disease was the most frequent comorbidity among children, and consequently, the most frequent surgical procedure was cardiac surgery.

The frequency of complications related to PICC use was 56.9% (95%CI 47.3;66.0). It was noted that device obstruction was the most frequent complication, followed by accidental removal and hyperemia during PICC insertion (Table 2). 

**Table 2:** Presentation of complications related to the use of Peripherally Inserted Central Venous Catheter in hospitalized children. Sorocaba, 2018-2022 (n=62)

	Complication
Variable	n (%)
Obstruction	33 (53.2)
Accidental withdrawal	11 (17.7)
Hyperemia	10 (16.1)
CLABSI^a^	3 (16.1)
Deep vein thrombosis	1 (1.6)
Death	4 (6.5)

^a^CLABSI – central line associated bloodstream infection.

It was observed that there is a greater chance of complications when the child is admitted to the neonatal pediatric intensive care unit (OR 5.45; 95%CI 1.20; 24.8) and when maintaining intravenous hydration (OR 3.71; 95%CI 1.02; 13.52) (Table 3). The other demographic, clinical and intravenous therapy variables were not related to the occurrence of complications.

**Table 3:** Association between demographic, clinical and intravenous therapy variables with the occurrence of complications related to the use of peripherally inserted central venous catheters in hospitalized children. Sorocaba, 2019-2022 (n=62)

Variables	Non-adjusted OR (95%CI)^a^	p-value^b^	Adjusted OR (95%CI)^a^	p-value^b^
Year		0.656		0.611
2019	1.00		1.00	
2020	0.64 (0.17; 2.44)		0.53 (0.13; 2.07)	
2021	0.54 (0.14; 2.03)		0.41 (0.10; 1.67)	
2022	0.39 (0.08; 1.84)		0.38 (0.08; 1.90)	
**Age range**		0.526		0.673
0-28 days	1.00		1.00	
29 days to 1 year	1.35 (0.56; 3.24)		1.23 (0.50; 3.01)	
Up to 2 years	1.87 (0.62; 5.65)		1.67 (0.54; 5.12)	
Gender		0.070		0.099
Male	1.00		1.00	
Female	2.07 (0.93; 4.61)		1.98 (0.88; 4.44)	
**Days with PICC^b^**		0.362		0.327
0-10	1.00		1.00	
11-20	0.55 (0.22; 1.39)		0.63 (0.24; 1.64)	
21 or more	0.57 (0.21; 1.57)		0.46 (0.16; 1.34)	
**Puncture site**		0.545		0.466
Upper limb	1.00		1.00	
Cephalic	2.82 (0.54; 14.81)		3.20 (0.58; 17.65)	
Jugular	0.90 (0.39; 2.09)		0.80 (0.33; 1.91)	
Lower limb	1.34 (0.29; 6.18)		1.40 (0.29; 6.71)	
**Puncture position**		0.120		0.168
Right	1.00		1.00	
Left	0.53 (0.24; 1.18)		0.56 (0.24; 1.28)	
**Superior vein**		0.582		0.654
Basilic	1.00		1.00	
Cephalic	0.55 (0.11; 2.86)		0.57 (0.09; 3.41)	
Axillary	0.92 (0.18; 4.58)		0.66 (0.12; 3.74)	
Cubital median	1.83 (0.48; 7.07)		1.74 (0.42; 7.12)	
**PICC Caliber^c^**		0.707		0.740
1.9 Fr	1.00		1.00	
2 Fr	0.90 (0.36; 2.28)		1.10 (0.42; 2.92)	
3 and 4 Fr	0.56 (0.13; 2.32)		0.66 (0.15; 2.92)	
**Lumen number**		0.923		0.617
1	1.00		1.00	
2 and 3	0.96 (0.42; 2.19)		1.26 (0.51; 3.12)	
**Puncture attempts**		0.716		0.733
1	1.00		1.00	
2	0.83 (0.23; 2.95)		0.88 (0.24; 3.22)	
3	0.46 (0.12; 2.95)		0.45 (0.11; 1.79)	
4 and 5	0.96 (0.28; 3.32)		0.90 (0.25; 3.21)	
**Place of hospitalization**		0.086		0.028
Pediatric ward	1.00		1.00	
NICU^d^/pediatric	3.44 (0.84; 14.11)		5.45 (1.20; 24.8)	
**Type of surgery**				
General	1.12 (0.49; 2.55)	0.782	1.67 (0.63; 4.41)	0.305
Cardiac	1.15 (0.53; 2.50)	0.723	1.17 (0.50; 2.76)	0.718
Neurological	2.23 (0.73; 6.77)	0.142	1.89 (0.55; 6.48)	0.311
Urological	0.31 (0.00; 4.01)	0.367^e^	0.33 (0.00; 4.48)	0.403^e^
Comorbidities				
Hypertension	0.76 (0.00; 29.56)	0.862^e^	0.61 (0.00; 23.76)	0.757^e^
Dyslipidemia	1.85 (0.14; ∞)	0.643^e^	1.05 (0.08; ∞)	0.973^e^
Heart disease	1.17 (0.52; 2.62)	0.707	1.27 (0.51; 3.17)	0.604
Immunosuppression	1.14 (0.18; 7.14)	0.885	1.48 (0.16; 18.72)	1.000^e^
Kidney disease	0.76 (0.02; ∞)	1,000^e^	0.98 (0.03; ∞)	1,000^e^
Pneumopathy	0.74 (0.18; 3.13)	0.684	0.71 (0.16; 3.17)	0.653
Down syndrome	4.17 (0.51; ∞)	0.201^e^	5.36 (0.64; ∞)	0.131^e^
**Use of inputs**				
Parenteral nutrition	2.18 (0.92; 5.20)	0.071	2.51 (0.97; 6.48)	0.057
Antibiotic	3.44 (0.84; 14.11)	0.071	2.61 (0.61; 11.18)	0.197
Antifungal	0.75 (0.05; 12.38)	0.843	0.77 (0.04; 13.70)	0.858
Hydration	3.43 (0.99; 11.95)	0.042	3.71 (1.02; 13.52)	0.047
Vesicants and irritants^e^	1.94 (0.84; 4.51)	0.120	1.94 (0.84; 4.51)	0.122
**Difficulty of access**	1.32 (0.03; ∞)	0.862^e^	0.83 (0.02; ∞)	1.000^e^

^a^The odds ratio (OR) was adjusted for sex and age group. ^a^95%CI – 95% confidence interval. ^b^Pearson’s chi-square test. ^c^PICC – peripherally inserted central venous catheter. ^d^NICU – neonatal intensive care unit. ^e^Exact logistic regression. eMedications considered: aminophylline, amiodarone, sodium bicarbonate, calcium chloride, potassium chloride, sodium chloride, diazepam, dobutamine, dopamine, epinephrine, phenylephrine, phenytoin, calcium gluconate, mannitol, metaraminol, norepinephrine, promethazine, thiopental, and vasopressin.

PICC obstruction was the most frequent complication – 30.3% (95%CI 22.3; 39.7). Being female (OR 2.93; 95%CI 1.24; 6.91), undergoing total parenteral nutrition (OR 2.97; 95%CI 1.11; 7.94) and having Down syndrome (OR 19.19; 95%CI 2.23; ∞) were factors associated with the occurrence of PICC obstruction (Table 4). When considering the other demographic, clinical and intravenous therapy variables, there was no association with the occurrence of obstructions.

**Table 4:** Association between demographic, clinical and intravenous therapy variables with the occurrence of obstruction related to the use of peripherally inserted ventral venous catheter in hospitalized children. Sorocaba, 2019-2022 (n=33)

	Obstruction			
Variable	n (%)	p-value^a^	OR^b^ adjusted (95%CI)^c^	p-value^a^
Year		0.137^d^		0.272
2019	1 (3.0)		1.00	
2020	16 (48.5)		7.07 (0.81; 61.74)	
2021	12 (36.4)		3.89 (0.42; 35.96)	
2022	4 (12,1)		4.28 (0.39; 46.99)	
**Age range**		0.582		0.665
0-28 days	9 (27.3)		1.00	
29 days to 1 year	15 (45,5)		0.83 (0.30; 2.28)	
Up to 2 years	9 (27.3)		1.35 (0.41; 4.41)	
Gender		0.011		0.014
Male	14 (42.4)		1.00	
Female	19 (57.6)		2.93 (1.24; 6.91)	
**Days with ^PICCe^**		0.216		0.219
0-10	19 (57.6)		1.00	
11-20	8 (24,2)		0.60 (0.21; 1.68)	
21 or more	6 (18.2)		0.37 (0.12; 1.20)	
**Puncture site**		0.682^d^		0.541
Upper limb	15 (45,5)		1.00	
Cephalic	4 (12,1)		3.04 (0.64; 14.39)	
Jugular	11 (33.3)		1.09 (0.41; 2.88)	
Lower limb	3 (9.1)		1.63 (0.32; 8.24)	
**Puncture position**		0.781		0.961
Right	16 (48.5)		1.00	
Left	13 (39.4)		1.02 (0.41; 2.56)	
**Superior vein**		0.623^d^		0.661
Basilic	5 (15,2)		1.00	
Cephalic	2 (6.1)		1.53 (0.19; 12.53)	
Axillary	2 (6.1)		0.86 (0.11; 6.42)	
Cubital median	6 (18.2)		236 (0.53; 10.57)	
**PICC caliber^e^**		0.256^d^		0.164
1.9 Fr	7 (21.2)		1.00	
2 Fr	25 (75.8)		2.06 (0.69; 6.18)	
3-4 Fr	1 (3.0)		0.37 (0.04; 3.75)	
**Lumen number**		0.997		0.350
1	10 (30.3)		1.00	
2-3	23 (69.7)		1.62 (0.59; 4.45)	
**Puncture attempts**		0.504^d^		0.487
1	26 (78.8)		1.00	
2	3 (9.1)		0.78 (0.18; 3.36)	
3	1 (3.0)		0.21 (0.02; 1.87)	
4-5	3 (9.1)		0.56 (0.13; 2.39)	
**Place of hospitalization**		0.720^d^		0.251
NICU^f^/pediatric	31 (93.9)		1.00	
Pediatric ward	2 (6.1)		0.37 (0.07; 2.01)	
**Type of surgery**				
General	9 (27.3)	0.560	0.90 (0.31; 2.65)	0.855
Cardiac	21 (63.6)	0.759	1.38 (0.53; 3.56)	0.506
Neurological	7 (21.2)	0.384	1.09 (0.32; 3.73)	0.889
Comorbidities				
Dyslipidemia	1 (3.0)	0.516^d^	1.18 (0.07; 20;89)	0.910
Heart disease	23 (69.7)	0.790	1.47 (0.55; 3.99)	0.445
Pneumopathy	2 (6.1)	1.000^d^	0.74 (0.13; 4.16)	0.732
Down syndrome	4 (12.1)	0.007^d^	19.19 (2.23; ∞)	0.006^g^
**Use of inputs**				
Parenteral nutrition	15 (45,5)	0.023	2.97 (1.11; 7.94)	0.030
Antibiotic	33 (100.0)	0.030^d^	4.21 (0.61; ∞)	0.164^g^
Hydration	30 (90.9)	0.751^d^	1.41 (0.34; 5.88)	0.634
**Vesicant and irritants**	25 (75.8)	0.522	1.36 (0.53; 3.45)	0.523

^a^Pearson’s chi-square test; ^b^Odds ratio (OR); ^c^95%CI – 95% confidence interval; ^d^Fisher’s exact test; ^e^Peripherally inserted central venous catheter; ^f^NICU – neonatal intensive care unit; ^g^Exact logistic regression.

## Discussion

More than half of pediatric patients had complications related to PICC use. Multivariate analysis revealed that patients in intensive care units with intravenous hydration were more likely to develop complications. Among the complications, device obstruction was the most frequent. Investigation of associated factors indicated that female children with Down syndrome and undergoing total parenteral nutrition had a greater chance of obstruction.

The research was conducted at an institution offering high and medium complexity care to patients with surgical needs. 26 of the 144 beds are for pediatric patients. In partnership with the Health Department, a goal was established to meet the demand for pediatric surgery in the region. All hospitalizations occur through the referral and counter-referral system, considering the severity and surgical specialty, with the Health Services Offer Regulation Center. Due to the fragility of the venous network in this population, intravenous catheters are essential for drug administration and to preserve the venous network in pediatric patients (7). However, studies on the use of central intravenous devices and their complications in children undergoing surgery are scarce.

This study had limitations regarding the recording of catheter insertion procedures. At the institution, treatment and procedures are recorded in the electronic medical record. However, there is no specific form with fields for recording PICC insertions and removals. Notes are made in open sections in the nursing records, both by the nurse responsible for the procedure and by the technical team that monitored the insertion of the device. It is important to highlight that, due to the lack of standardization in the notes on the insertion and removal of the device, the outcome assessment may present variability during the analysis of the results. This variability was found in Sweden between March and April 2022. Practices for inserting and maintaining intravenous devices, when not well documented, can generate inaccuracy in the collection of related information (8,9). In Australia, among 220 adult patients evaluated, only peripheral venous access was recorded on a specific form, with dates of insertion, removal, and replacement. There was no space on the form for the same information regarding PICC and central venous catheter (9). 

Despite the limitation found in the insertion and removal records of the devices in this study, it was possible to identify the dispensing of the PICC to the patient through the institution’s cost center. This approach has proven effective, due to the organized structure of the cost center, which keeps detailed records of the materials intended for patients. It is worth noting that more complications (cases) were found than the absence of complications (control). Probably, the hospital’s profile in caring for serious pediatric patients presupposes that they have a greater chance of complications than would be observed in another institution. To overcome this limitation, adjusted logistic regressions, including exact logistic regression, were performed to deal with small sample sizes.

In this study, the reasons for PICC removal included obstruction, accidental removal, presence of hyperemia, bloodstream infection associated with the central line, deep vein thrombosis, and death. Although the procedure is considered safe, complications can arise during its use in all age groups. National and international research has demonstrated similarities in the complications found in this study (3,10,11). These complications can interrupt treatment and interfere with clinical progress, prolong hospitalization, compromise patient safety and quality of life, increase healthcare costs and overload the healthcare team, as they require continuous attention from professionals and can have an impact on the Brazilian Unified Health System (12).

Among children, those who remained hospitalized in the intensive care unit had a greater chance of complications. It is important to mention that there was a higher frequency of cardiac surgeries, and the most frequent comorbidity was heart disease. The complexity of the surgeries performed, and the prolonged hospital stay increased the chances of complications. Still regarding the multivariate analysis, children using continuous hydration also had a greater chance of developing complications. This data drew attention to the maintenance of the PICC, since the use of intravenous hydration does not replace the saline infusion solution and device maintenance protocols (13).

PICC obstruction was the most frequent complication in this study. Similar findings occurred in national and international studies. In a public institution specialized in pediatric oncology in the city of Rio de Janeiro, obstruction occurred in 64.4% of cases (11). In 2023, in Indonesia, 40.0% of children presented complications, and among the complications, occlusion was the most frequent (14).

When studying the factors associated with obstruction, this study identified that female children with Down syndrome and undergoing total parenteral nutrition had a greater chance of device obstruction. In the literature, sex is addressed more broadly, without direct association with the obstruction or complication factor (15,17). No studies were found that Down syndrome directly associates the obstruction factor in peripherally or centrally inserted catheters. However, studies were identified that reported the difficulty of inserting intravenous access in younger children, especially those with complex diseases and the presence of heart disease (18). Among the four children with Down syndrome, three had heart disease, which may have influenced the observed outcomes. 

In China, one-third of pediatric cardiac patients had device obstruction (19). Such factors may represent a confounding bias in this study, especially in the association between heart disease and increased complications. Although no studies like this one were found, it is believed that the small sample size may have influenced this result. A relevant aspect is that all children with Down syndrome were admitted to the intensive care unit and received intravenous hydration, factors that, in this analysis, demonstrated a greater chance of complications. It was also observed that two of these children made use of total parenteral nutrition. These data indicated that the total parenteral nutrition is associated with a greater chance of obstruction.

Regarding the use of total parenteral nutrition and the obstruction factor, in 2019, among the adverse events related to parenteral drug administration, more than half of the patients in the study presented device obstruction. It was not possible to accurately determine the exact proportion of complications directly associated with total parenteral nutrition (20). It has been suggested that the association of total parenteral nutrition with other medications, such as antibiotics, increases the risk of occlusion due to the progressive deposition of microcrystals along the catheter wall (20,21). In this study, almost all children continued to use antibiotics. 

Data from 2019 indicated that patients who received total parenteral nutrition as a primary source of nutrition presented an elevated risk of infectious and non-infectious complications related to the catheter, including obstruction of central devices (22). In Canada in 2021, the most common complications in pediatric patients using total parenteral nutrition were central line-associated bloodstream infection and obstruction (23). The number of bloodstream infections associated with the central line was much lower when compared to obstruction (23). Other factors may be associated with obstruction of central devices, such as ongoing education of the nursing team, knowledge and skills of nurses, saline infusion solution and method, and insertion techniques, which were not evaluated in this research (15,20).

In pediatric patients, the use of a safe intravenous device is essential for effective treatment. PICC has been widely used, becoming a safe alternative for venous access in this group in the hospital environment (3,24).

In the hospital context, it is important that care records and the insertion of intravenous devices are properly recorded in the medical record. This information is essential for patient safety and for detecting failures in device care and maintenance (25). Maintaining structured and standardized records in electronic medical records increases the accuracy and quality of central catheter records, such as PICC (8,26). This practice should be adopted before and after the procedure, as gaps in records represent a challenge to ensuring patient safety (8).

Standardization of materials for PICC insertion is essential to ensure the quality and safety of the procedure. Its absence may lead to the acquisition of inferior materials and the need for additional training of nursing staff. At the institution where the study was conducted, standardization did not occur due to limitations in the purchasing system. The available catheters were made of silicone and had no valves. The inclusion of valves, regardless of position, can prevent blood reflux when disconnecting the catheter and reduce the risk of occlusion. It is important to note that other precautions associated with the presence of the valve must be taken, such as the infusion of saline solution after the administration of intermittent medications and blood products (27).

During the research, it was observed that the complications identified in this study were common in pediatric hospitals in developed nations and in developing countries (28,29). Among the associated factors in the study, obstruction may be related to the use of total parenteral nutrition. It is the nurse’s responsibility to ensure maintenance, permeability, and an adequate route of administration for patients using total parenteral nutrition (30). Furthermore, maintaining continuous intravenous hydration in the PICC does not replace the standardization of the saline infusion solution protocol, which was not available at the institution. 

It is concluded that patients in intensive care have a greater chance of complications due to the criticality of cases, prolonged hospitalization time and comorbidities. Maintaining intravenous hydration also demonstrated a greater chance of complications. Identifying factors associated with complications of PICC insertion in this study may assist health services in prioritizing the procedure or a more intensive monitoring when insertion is necessary. Such actions may benefit patients and improve the performance of the Unified Health System.

## Data Availability

Data is not available, as access is restricted to the hospital.
