# Supplementary material for: Factors associated with complications related to the use of peripherally inserted central venous catheter in hospitalized children: a case-control study, Sorocaba, Brazil, 2018-2022
Source: Epidemiol Serv Saude. 2025 Sep 8;34:e20240134. doi: 10.1590/S2237-96222025v34e20240134.en (PMC12435953; doi:10.1590/S2237-96222025v34e20240134.en)
Supplement: Translation

# Translation: Fatores associados a complicações relacionadas ao uso de cateter venoso central de inserção periférica em crianças hospitalizadas: estudo caso-controle, Sorocaba, 2018-2022

**DOI:** 10.1590/S2237-96222025v34e20240134.pt

**Authors:** Mayara Amancio de Souza, Luciane Cruz Lopes, Marcus Tolentino Silva

**Affiliations:** 1 Universidade de Sorocaba, Programa de Pós-Graduação em Ciências Farmacêuticas, Sorocaba, SP, Brasil; 2 Universidade Federal de Brasília, Departamento de Saúde Coletiva, Brasília, DF, Brasil

**Keywords:** Obstrução do Cateter, Cateteres Venosos Centrais, Cateterismo Periférico, Pediatria, Estudos de Casos e Controles

## Abstract

**Objetivo:**

Verificar fatores associados a complicações relacionadas ao uso de cateter venoso central de inserção periférica em crianças submetidas a procedimentos cirúrgicos.

**Métodos:**

Tratou-se de estudo caso-controle, realizado em hospital público de Sorocaba, com ênfase em procedimentos cirúrgicos, entre 2018 e 2022. O prontuário eletrônico foi utilizado como fonte de informação. Foram calculadas as razões de chance (*odds ratio*, OR) e os intervalos de confiança de 95% (IC95%).

**Resultados:**

Incluíram-se na pesquisa 109 crianças de até 2 anos, 11 meses e 29 dias, das quais 62 eram casos, e 47, controles. A frequência de complicações relacionadas ao uso do cateter venoso central de inserção periférica foi 56,9% (IC95% 47,3; 66,0), e as mais frequentes foram obstrução (30,3%) (IC95% 22,3; 39,7), retirada acidental (10,1%) e hiperemia (9,2%). Houve maior chance de complicação quando a criança se manteve na unidade de cuidados intensivos neonatal pediátrico (OR 5,45; IC95% 1,20; 24,8) e ao manter hidratação intravenosa (OR 3,71; IC95% 1,02; 13,52). Crianças do sexo feminino (OR 2,93; IC95% 1,24; 6,91), em uso de nutrição parenteral total (OR 2,97; IC95% 1,11; 7,94) e com síndrome de Down (OR 19,19; IC95% 2,23; ∞) apresentaram maior chance de obstrução.

**Conclusão:**

Crianças em cuidados intensivos e sob hidratação intravenosa apresentaram maior chance de complicações. Compreender os fatores associados à complicação beneficia pacientes, as instituições de saúde e o Sistema Único de Saúde.

**Aspectos éticos**

A presente pesquisa respeitou os princípios éticos, obtendo os seguintes dados de aprovação:

Comitê de ética em pesquisa: Universidade de Sorocaba

Número do parecer: 5.973.039

Data de aprovação: 29/3/2023

Certificado de apresentação de apreciação ética: 66973123.6.0000.5500

Registro do consentimento livre e esclarecido: Dispensado.

## Introdução

Nos pacientes pediátricos, o procedimento invasivo mais comum durante a hospitalização é a inserção de cateteres intravenosos (1). A maioria das crianças, especialmente as submetidas a cirurgias, necessita de acesso vascular para administrar medicamentos e realizar exames. Esses dispositivos são essenciais para tratamentos como hidratação, infusão de antibióticos, drogas vesicantes, hemocomponentes, nutrição parenteral total e medicamentos para equilíbrio hidroeletrolítico (1,2). Em neonatos e lactantes, garantir o acesso intravenoso ainda é um desafio devido às dificuldades na punção e manutenção, pois a rede venosa apresenta menor calibre e visibilidade nesse período (3). 

O cateter venoso central de inserção periférica, do inglês *peripherally inserted central catheter* (PICC), é uma opção segura e cada vez mais usada em pacientes pediátricos, com vantagens como terapia intravenosa prolongada, preservação da rede venosa, redução da dor e estresse e administração de soluções hipertônicas. Comparado a outros cateteres centrais, oferece menor custo, menor risco de infecção, menor incidência de pneumotórax e maior mobilidade ao paciente (3,4). O cateter venoso central de inserção periférica pode ser inserido no leito por enfermeiros capacitados (4).

Apesar dos benefícios, o PICC não é isento de complicações. Entre os pacientes pediátricos, as complicações mais comuns incluem trombose, obstrução, infiltração intravenosa, infecção de corrente sanguínea e complicações potencialmente fatais, como derrame pleural, tamponamento pericárdico e derrame pericárdico (5).

No Brasil, poucos estudos avaliaram o uso do PICC no Sistema Único de Saúde em pacientes cirúrgicos pediátricos. Por meio do estudo caso-controle, é possível compreender os fatores associados à ocorrência de complicações em crianças nessa faixa etária. A compreensão dos fatores associados pode favorecer ações direcionadas à redução de complicações nas instituições de saúde. Essas iniciativas podem promover benefícios aos pacientes e ao Sistema Único de Saúde, com a otimização de recursos e a melhora da qualidade da assistência prestada. O objetivo desta pesquisa é verificar os fatores associados à ocorrência de complicações relacionadas ao uso do PICC em crianças submetidas a procedimentos cirúrgicos.

## Métodos

### Delineamento

Tratou-se de estudo caso-controle. Todas as crianças foram submetidas à inserção do PICC. O grupo casos foi constituído pelos pacientes que tiveram algum tipo de complicação. Considerou-se como complicação o desfecho primário deste estudo: a remoção não eletiva do dispositivo, tais como retirada acidental, óbito, hiperemia, obstrução, trombose venosa profunda e infecção durante o período de hospitalização. 

O grupo controle foi definido como: os pacientes que usaram o PICC no mesmo período e não tiveram complicações; as altas hospitalares; os desvios de qualidade, os quais foram identificados quando o PICC não atendeu aos parâmetros de qualidade; as falhas na adaptação dos conectores; além daqueles que necessitaram de contrarreferência com o PICC inserido, mas sem acompanhamento à retirada do dispositivo. 

### Contexto

A pesquisa foi realizada no Hospital Regional de Sorocaba “Dr. Adib Domingos Jatene”, hospital público localizado em Sorocaba, São Paulo, após a aprovação dos comitês de ética da instituição e da Universidade de Sorocaba. Gerenciado pela Associação Paulista para o Desenvolvimento da Medicina, em parceria com a Secretaria de Estado da Saúde de São Paulo, o hospital é referência terciária em especialidades cirúrgicas como ortopedia e traumatologia, cirurgia do aparelho circulatório, cirurgia geral e trauma, neurocirurgia, cirurgia pediátrica, e clínica médica com ênfase em infarto agudo do miocárdio e acidente vascular cerebral. 

A instituição dispõe de 144 leitos, dos quais 26 são pediátricos. Destes, 16 são para a enfermaria de pediatria cirúrgica, e 10, para a unidade de cuidados intensivos neonatal pediátrico cirúrgico, com ênfase em cardiopatias congênitas e cirurgias pediátricas. Em maio de 2024, a instituição obteve a recertificação de Acreditação com Excelência, nível 3, pela Organização Nacional de Acreditação. A recertificação da Acreditação com Excelência é a reavaliação realizada por uma entidade acreditadora para confirmar que uma instituição de saúde mantém elevados padrões de qualidade e segurança no atendimento aos pacientes (6).

A compra do PICC e dos demais materiais no hospital é realizada de forma consolidada. Após a consolidação dos pedidos, os lançamentos e as publicações são feitas para todos os fornecedores cadastrados no Portal Eletrônico. O critério de seleção é o menor preço, desde que atenda à especificação técnica. O produto não é adquirido em casos de restrição de marca pela Agência Nacional de Vigilância Sanitária ou bloqueio pelo Comitê Técnico de Qualificação de Fornecedores da instituição. Essa logística de aquisição impede a padronização do PICC quanto à qualidade dos materiais (silicone ou poliuretano), ao número de lúmens, às medidas em French (Fr) e à presença de microintrodutor para punções diretas ou guiadas por ultrassom.

### Participantes

Os participantes da pesquisa foram crianças, submetidas a procedimentos cirúrgicos e que utilizaram o PICC na instituição entre 2018 e 2022. A amostra incluiu 109 crianças, subdivididas entre: 0 e 28 dias; 29 dias a 1 ano; e até 2 anos, 11 meses e 29 dias de idade. Foram excluídas crianças com mais de 3 anos, registros de passagens malsucedidas do PICC e casos em que, apesar da solicitação para inserção do PICC, realizou-se a passagem do cateter venoso central.

### Variáveis

Para a caracterização da amostra, foram calculadas as frequências e medidas de tendência central e a dispersão de algumas variáveis. Considerou-se o ano de inserção do PICC durante o período da pandemia de coronavírus 2019. As variações demográficas incluíram sexo (masculino, feminino) e idade (em meses). As variáveis ​​clínicas compreenderam a presença de comorbidades (hipertensão, dislipidemia, cardiopatia, imunossupressão, doença renal, pneumopatia e síndrome de Down), tipos de cirurgias realizadas (geral, cardíaca, neurológica, urologia), modalidades de terapia infusional (nutrição parenteral, medicamentos) e os locais de inserção do PICC (membros superiores, membros inferiores, veias jugulares, femorais, axilar, cefálica). Quanto às variáveis ​​relacionadas ao PICC, foram analisados ​​o número de tentativas de inserção, o tempo de permanência do cateter, o tipo de punção (direta, guiada por ultrassom), os calibres utilizados, o motivo da retirada do PICC e a ocorrência de complicações.

### Fontes de dados e mensuração

Os dados foram coletados em 2023. Como fonte de informação, utilizaram-se o prontuário eletrônico e o sistema operacional de gestão hospitalar durante o período de internação entre 2018 e 2022.

Para a extração dos dados, foi elaborado um questionário pela autora com a ferramenta KoBoToolbox. O registro das informações ocorreu por meio de dispositivos eletrônicos móveis. Durante a coleta das informações, realizada pela autora da pesquisa, os dados foram constantemente verificados por meio da conferência das informações carregadas na plataforma do KoBoToolbox, a fim de identificar e corrigir eventuais inconsistências, como a falha de carregamento dos dados coletados. 

### Controle de viés

Para reduzir o viés de informação em relação à coleta de dados, foi possível identificar a dispensação do PICC ao paciente por meio da central de custos da instituição. Com base nessa informação, foi realizada a conferência sistemática dos dados no prontuário eletrônico, a partir da data em que o PICC foi dispensado, o que possibilitou a correção de eventuais inconsistências.

Para mitigar o viés de confusão decorrente da gravidade dos pacientes, uma vez que o perfil do hospital atende aqueles com maior risco de complicações, realizaram-se análises multivariadas considerando a idade como indicador substituto de prognóstico.

### Tamanho do estudo

Por meio do prontuário eletrônico, buscou-se a totalidade das crianças com uso de PICC na instituição, assim não foi realizado o cálculo de tamanho de amostra. Considerando o número de casos e controles disponíveis, calculou-se o poder como nível de confiança a 95%.

### Métodos estatísticos

As variáveis categóricas foram analisadas usando teste qui-quadrado, ou exato de Fisher quando apropriado. Consideraram-se estatisticamente significativos p-valor menores do que 0,005%.

Para investigar as medidas de associação entre as complicações mais frequentes, foram utilizados modelos de regressão logística univariada e multivariada. Os resultados foram apresentados como razão de chances (*odds ratio*, OR) ajustados por faixa etária e sexo, com intervalos de confiança de 95% (IC95%). Quando necessário, procedeu-se o modelo de regressão logística exata para lidar melhor com excesso de resultados positivos para o desfecho. A mesma rotina foi aplicada com a complicação mais frequente no estudo. Todos os cálculos foram executados no STATA 14.2.

## Resultados

Foram analisados 392 prontuários com solicitação de inserção de PICC e efetivadas 125 inserções (Figura 1). Destas, 109 crianças foram incluídas na pesquisa, das quais identificaram-se 62 casos e 47 controles. O poder do estudo foi estimado em 89,0% considerando como fator de exposição a internação em unidade de cuidado intensivo pediátrico.

**Figura 1:** Fluxo de inclusão e seleção de pacientes no estudo. Sorocaba, 2018-2022

A amostra desta pesquisa foi composta por 109 indivíduos que fizeram uso do PICC (Tabela 1). O período pandêmico não influenciou os resultados do estudo. A maior frequência de inserção do PICC ocorreu nas crianças de 29 dias a 1 ano. O sexo masculino representou a maioria das inserções em comparação ao sexo feminino. A maior frequência quanto ao tempo e à permanência do PICC foi entre 0 e 10 dias. O local de escolha de punção mais frequente ocorreu nos membros superiores e, em relação à lateralidade de posição da punção, no lado direto. O cateter triplo lúmen foi incluído com o cateter de duplo lúmen, devido à ocorrência de apenas uma observação.

**Tabela 1:** Caracterização demográfica, clínica e da terapia intravenosa das crianças dos grupos caso e controle. Sorocaba, 2019-2022 (n=109)

	Amostra	Casos	Controle	
Variável	n (%)	n=62	n=47	p-valor^a^
Ano				0,661
2019	13 (11,9)	9	4	
2020	39 (35,8)	23	16	
2021	42 (38,5)	23	19	
2022	15 (13,80)	7	8	
**Faixa etária**				0,528
0-28 dias	32 (29,4)	16	16	
29 dias a 1 ano	54 (49,5)	31	23	
Até 2 anos	23 (21,1)	15	8	
Sexo				0,072
Masculino	66 (60,6)	33	33	
Feminino	43 (39,5)	29	14	
**Nº dias PICC** ^b^				0,366
0-10	46 (45,1)	31	15	
11-20	32 (31,4)	17	15	
21 ou mais	24 (23,5)	13	11	
**Sítio de punção**				0,595^c^
Membro superior	56 (51,4)	31	25	
Cefálica	9 (8,3)	7	2	
Jugular	36 (33,0)	19	17	
Membro inferior	8 (7,3)	5	3	
**Posição da punção**				0,121
Direita	53 (53,0)	33	20	
Esquerda	47 (47,0)	22	25	
**Veia superior**				0,589^c^
Basílica	23 (42,6)	12	11	
Cefálica	8 (14,8)	3	5	
Axilar	8 (14,8)	4	4	
Mediana cubital	15 (27,8)	10	5	
**Calibre do PICC** ^b^				0,723^c^
1,9 Fr	25 (22,9)	15	10	
2 Fr	73 (67,0)	42	31	
3-4 Fr	11 (10,0)	5	6	
**Nº de lúmen**				0,923
1	33 (30,3)	19	14	
2-3	76 (76,0)	43	33	
**Tentativas de punção**				0,707^c^
1	76 (69,7)	45	31	
2	11(10,0)	6	5	
3	10 (9,2)	4	6	
4-5	12 (11,0)	7	5	
**Local de internação**				0,097^c^
Enfermaria pediátrica	10 (9,1)	3	7	
UCIN^d^/pediátrica	99 (99,8)	59	40	
**Tipo de cirurgia**				
Geral	34 (31,2)	20	14	0,783
Cardíaca	67 (61,5)	39	28	0,724
Neurológica	18 (16,5)	13	5	0,150
Urológica	2 (1,8)	0	2	0,184^c^
Comorbidades				
Hipertensão	1 (0,9)	0	1	0,431^c^
Dislipidemia	2 (1,8)	2	0	0,505^c^
Cardiopatia	74 (67,9)	43	31	0,707
Imunossupressão	5 (4,6)	3	2	1,000^c^
Doença renal	1 (0,9)	1	0	1,000^c^
Pneumopatia	8 (7,3)	4	4	0,724^c^
Síndrome de Down	4 (3,7)	4	0	0,132^c^
**Uso de insumos**				
Nutrição parenteral	33 (30,3)	23	10	0,075
Antibiótico	99 (99,8)	59	40	0,072
Antifúngico	2 (1,8)	1	1	1,000^c^
Hidratação	96 (88,1)	58	38	0,043
Vesicante e irritantes^e^	78 (71,6)	48	30	0,119
**Dificuldade de acesso**	108 (99,1)	62	46	0,431^c^

^a^Teste qui-quadrado de Pearson; ^b^PICC – cateter venoso central de inserção periférica; ^c^Teste exato de Fisher; e ^d^UCIN – unidade de cuidado intensivo neonatal; ^e^Medicamentos considerados: aminofilina, amiodarona, bicarbonato de sódio, cloreto de cálcio, cloreto de potássio, cloreto de sódio, diazepam, dobutamina, dopamina, epinefrina, fenilefrina, fenitoína, gluconato de cálcio, manitol, metaraminol, norepinefrina, prometazina, tiopental e vasopressina.

Todas as punções foram realizadas de forma direta, sem auxílio do ultrassom. A veia basílica foi a mais frequente na punção dos membros superiores. Quanto às características do PICC, todos eram sem ponta valvar e de silicone, e o calibre mais utilizado foi 2 French e o cateter de duplo lúmen. O local com maior frequência de inserção de PICC foi a unidade de cuidados intensivos neonatal pediátrico, e os dados demonstraram que a maioria dos profissionais obteve êxito na primeira tentativa de punção. A cardiopatia teve a maior frequência entre as comorbidades apresentadas pelas crianças, e, consequentemente, o procedimento cirúrgico com maior frequência foram as cirurgias cardíacas.

A frequência de complicações relacionadas ao uso do PICC foi 56,9% (IC95% 47,3; 66,0). Notou-se que a obstrução do dispositivo apresentou a maior frequência, seguido de retirada acidental e hiperemia na inserção do PICC (Tabela 2). 

**Tabela 2:** Apresentação das complicações relacionadas ao uso de Cateter Venoso Central de Inserção Periférica em crianças hospitalizadas. Sorocaba, 2018-2022 (n=62)

	Complicação
Variável	n (%)
Obstrução	33 (53,2)
Retirada acidental	11 (17,7)
Hiperemia	10 (16,1)
CLABSI^a^	3 (16,1)
Trombose venosa profunda	1 (1,6)
Óbito	4 (6,5)

^a^CLABSI – infecção de corrente sanguínea associada à linha central.

Observou-se que há maior chance de complicação quando a criança está internada na unidade de cuidados intensivos neonatal pediátrica (OR 5,45; IC95% 1,20; 24,8) e ao manter hidratação intravenosa (OR 3,71; IC95% 1,02; 13,52) (Tabela 3). As demais variáveis demográficas, clínicas e da terapia intravenosa não tiveram relação com a ocorrência de complicações.

**Tabela 3:** Associação entre variáveis demográficas, clínicas e da terapia intravenosa com a ocorrência de complicações relacionadas ao uso de cateter venoso central de inserção periférica em crianças hospitalizadas. Sorocaba, 2019-2022 (n=62)

Variáveis	OR não ajustado (IC95%)^a^	p-valor^b^	OR ajustado (IC95%)^a^	p-valor^b^
Ano		0,656		0,611
2019	1,00		1,00	
2020	0,64 (0,17; 2,44)		0,53 (0,13; 2,07)	
2021	0,54 (0,14; 2,03)		0,41 (0,10; 1,67)	
2022	0,39 (0,08; 1,84)		0,38 (0,08; 1,90)	
**Faixa etária**		0,526		0,673
0-28 dias	1,00		1,00	
29 dias a 1 ano	1,35 (0,56; 3,24)		1,23 (0,50; 3,01)	
Até 2 anos	1,87 (0,62; 5,65)		1,67 (0,54; 5,12)	
Sexo		0,070		0,099
Masculino	1,00		1,00	
Feminino	2,07 (0,93; 4,61)		1,98 (0,88; 4,44)	
**Nº dias PICC^c^**		0,362		0,327
0-10	1,00		1,00	
11-20	0,55 (0,22; 1,39)		0,63 (0,24; 1,64)	
21 ou mais	0,57 (0,21; 1,57)		0,46 (0,16; 1,34)	
**Sítio de punção**		0,545		0,466
Membro superior	1,00		1,00	
Cefálica	2,82 (0,54; 14,81)		3,20 (0,58; 17,65)	
Jugular	0,90 (0,39; 2,09)		0,80 (0,33; 1,91)	
Membro inferior	1,34 (0,29; 6,18)		1,40 (0,29; 6,71)	
**Posição da punção**		0,120		0,168
Direita	1,00		1,00	
Esquerda	0,53 (0,24; 1,18)		0,56 (0,24; 1,28)	
**Veia superior**		0,582		0,654
Basílica	1,00		1,00	
Cefálica	0,55 (0,11; 2,86)		0,57 (0,09; 3,41)	
Axilar	0,92 (0,18; 4,58)		0,66 (0,12; 3,74)	
Mediana cubital	1,83 (0,48; 7,07)		1,74 (0,42; 7,12)	
**Calibre do PICC** ^c^		0,707		0,740
1,9 Fr	1,00		1,00	
2 Fr	0,90 (0,36; 2,28)		1,10 (0,42; 2,92)	
3 e 4 Fr	0,56 (0,13; 2,32)		0,66 (0,15; 2,92)	
**Nº de lúmen**		0,923		0,617
1	1,00		1,00	
2 e 3	0,96 (0,42; 2,19)		1,26 (0,51; 3,12)	
**Tentativas de punção**		0,716		0,733
1	1,00		1,00	
2	0,83 (0,23; 2,95)		0,88 (0,24; 3,22)	
3	0,46 (0,12; 2,95)		0,45 (0,11; 1,79)	
4 e 5	0,96 (0,28; 3,32)		0,90 (0,25; 3,21)	
**Local de internação**		0,086		0,028
Enfermaria pediátrica	1,00		1,00	
UCIN^d^/pediátrica	3,44 (0,84; 14,11)		5,45 (1,20; 24,8)	
**Tipo de cirurgia**				
Geral	1,12 (0,49; 2,55)	0,782	1,67 (0,63; 4,41)	0,305
Cardíaca	1,15 (0,53; 2,50)	0,723	1,17 (0,50; 2,76)	0,718
Neurológica	2,23 (0,73; 6,77)	0,142	1,89 (0,55; 6,48)	0,311
Urológica	0,31 (0,00; 4,01)	0,367^e^	0,33 (0,00; 4,48)	0,403^e^
Comorbidades				
Hipertensão	0,76 (0,00; 29,56)	0,862^e^	0,61 (0,00; 23,76)	0,757^e^
Dislipidemia	1,85 (0,14; ∞)	0,643^e^	1,05 (0,08; ∞)	0,973^e^
Cardiopatia	1,17 (0,52; 2,62)	0,707	1,27 (0,51; 3,17)	0,604
Imunossupressão	1,14 (0,18; 7,14)	0,885	1,48 (0,16; 18,72)	1,000^e^
Doença renal	0,76 (0,02; ∞)	1,000^e^	0,98 (0,03; ∞)	1,000^e^
Pneumopatia	0,74 (0,18; 3,13)	0,684	0,71 (0,16; 3,17)	0,653
Síndrome de Down	4,17 (0,51; ∞)	0,201^e^	5,36 (0,64; ∞)	0,131^e^
**Uso de insumos**				
Nutrição parenteral	2,18 (0,92; 5,20)	0,071	2,51 (0,97; 6,48)	0,057
Antibiótico	3,44 (0,84; 14,11)	0,071	2,61 (0,61; 11,18)	0,197
Antifúngico	0,75 (0,05; 12,38)	0,843	0,77 (0,04; 13,70)	0,858
Hidratação	3,43 (0,99; 11,95)	0,042	3,71 (1,02; 13,52)	0,047
Vesicante e irritantes^e^	1,94 (0,84; 4,51)	0,120	1,94 (0,84; 4,51)	0,122
**Dificuldade de acesso**	1,32 (0,03; ∞)	0,862^e^	0,83 (0,02; ∞)	1,000^e^

^a^A razão de chances (*odds ratio*, OR) foi ajustada por sexo e faixa etária. ^a^IC95% – intervalo de confiança de 95%; ^b^Teste qui-quadrado de Pearson; ^c^PICC – cateter venoso central de inserção periférica; e ^d^UCNI – unidade de cuidado intensivo neonatal; ^e^Regressão logística exata; ^e^Medicamentos considerados: aminofilina, amiodarona, bicarbonato de sódio, cloreto de cálcio, cloreto de potássio, cloreto de sódio, diazepam, dobutamina, dopamina, epinefrina, fenilefrina, fenitoína, gluconato de cálcio, manitol, metaraminol, norepinefrina, prometazina, tiopental e vasopressina.

A obstrução do PICC entre as complicações demonstrou maior frequência – 30,3% (IC95% 22,3; 39,7). Ser do sexo feminino (OR 2,93; IC95% 1,24; 6,91), fazer uso de nutrição parenteral total (OR 2,97; IC95% 1,11; 7,94) e ter síndrome de Down (OR 19,19; IC95% 2,23; ∞) foram fatores associados à ocorrência de obstrução no PICC (Tabela 4). Ao considerar as demais variáveis demográficas, clínicas e da terapia intravenosa, não houve associação de ocorrência de obstruções.

**Tabela 4:** Associação entre variáveis demográficas, clínicas e da terapia intravenosa com a ocorrência de obstrução relacionada ao uso de cateter venoso ventral de inserção periférica em crianças hospitalizadas. Sorocaba, 2019-2022 (n=33)

	Obstrução			
Variável	n (%)	p-valor^a^	OR^b^ ajustado (IC95%)^c^	p-valor^a^
Ano		0,137^d^		0,272
2019	1 (3,0)		1,00	
2020	16 (48,5)		7,07 (0,81; 61,74)	
2021	12 (36,4)		3,89 (0,42; 35,96)	
2022	4 (12,1)		4,28 (0,39; 46,99)	
**Faixa etária**		0,582		0,665
0-28 dias	9 (27,3)		1,00	
29 dias a 1 ano	15 (45,5)		0,83 (0,30; 2,28)	
Até 2 anos	9 (27,3)		1,35 (0,41; 4,41)	
Sexo		0,011		0,014
Masculino	14 (42,4)		1,00	
Feminino	19 (57,6)		2,93 (1,24; 6,91)	
**Nº dias PICC^e^**		0,216		0,219
0-10	19 (57,6)		1,00	
11-20	8 (24,2)		0,60 (0,21; 1,68)	
21 ou mais	6 (18,2)		0,37 (0,12; 1,20)	
**Sítio de punção**		0,682^d^		0,541
Membro superior	15 (45,5)		1,00	
Cefálica	4 (12,1)		3,04 (0,64; 14,39)	
Jugular	11 (33,3)		1,09 (0,41; 2,88)	
Membro inferior	3 (9,1)		1,63 (0,32; 8,24)	
**Posição da punção**		0,781		0,961
Direita	16 (48,5)		1,00	
Esquerda	13 (39,4)		1,02 (0,41; 2,56)	
**Veia superior**		0,623^d^		0,661
Basílica	5 (15,2)		1,00	
Cefálica	2 (6,1)		1,53 (0,19; 12,53)	
Axilar	2 (6,1)		0,86 (0,11; 6,42)	
Mediana cubital	6 (18,2)		2.36 (0,53; 10,57	
**Calibre do PICC** ^e^		0,256^d^		0,164
1,9 Fr	7 (21,2)		1,00	
2 Fr	25 (75,8)		2,06 (0,69; 6,18)	
3-4 Fr	1 (3,0)		0,37 (0,04; 3,75)	
**Nº de lúmen**		0,997		0,350
1	10 (30,3)		1,00	
2-3	23 (69,7)		1,62 (0,59; 4,45)	
**Tentativas de punção**		0,504^d^		0,487
1	26 (78,8)		1,00	
2	3 (9,1)		0,78 (0,18; 3,36)	
3	1 (3,0)		0,21 (0,02; 1,87)	
4-5	3 (9,1)		0,56 (0,13; 2,39)	
**Local de internação**		0,720^d^		0,251
UCIN^f^/pediátrica	31 (93,9)		1,00	
Enfermaria pediátrica	2 (6,1)		0,37 (0,07; 2,01)	
**Tipo de cirurgia**				
Geral	9 (27,3)	0,560	0,90 (0,31; 2,65)	0,855
Cardíaca	21 (63,6)	0,759	1,38 (0,53; 3,56)	0,506
Neurológica	7 (21,2)	0,384	1,09 (0,32; 3,73)	0,889
Comorbidades				
Dislipidemia	1 (3,0)	0,516^d^	1,18 (0,07; 20,89)	0,910
Cardiopatia	23 (69,7)	0,790	1,47 (0,55; 3,99)	0,445
Pneumopatia	2 (6,1)	1,000^d^	0,74 (0,13; 4,16)	0,732
Síndrome de Down	4 (12,1)	0,007^d^	19,19 (2,23; ∞)	0,006^g^
**Uso de insumos**				
Nutrição parenteral	15 (45,5)	0,023	2,97 (1,11; 7,94)	0,030
Antibiótico	33 (100,0)	0,030^d^	4,21 (0,61; ∞)	0,164^g^
Hidratação	30 (90,9)	0,751^d^	1,41 (0,34; 5,88)	0,634
**Vesicante e irritantes**	25 (75,8)	0,522	1,36 (0,53; 3,45)	0,523

^a^Teste qui-quadrado de Pearson; ^b^Razão de chances (*odds ratio*, OR); ^c^IC95% – intervalo de confiança de 95%; ^d^Teste exato de Fisher; ^e^Cateter venoso central de inserção periférica; ^f^UCIN – unidade de cuidado intensivo neonatal; e ^g^Regressão logística exata.

## Discussão

Mais da metade dos pacientes pediátricos apresentaram complicações relacionadas ao uso do PICC.

A análise multivariada revelou que pacientes em unidade de cuidados intensivos e com hidratação intravenosa apresentaram maior chance de desenvolvê-las. Entre as complicações, a obstrução do dispositivo foi a mais frequente. A investigação dos fatores associados indicou que crianças do sexo feminino, com síndrome de Down e em uso de nutrição parenteral total apresentaram maior chance de obstrução.

A pesquisa foi conduzida em uma instituição que oferece atendimento de alta e média complexidade a pacientes com necessidades cirúrgicas. Dos 144 leitos, 26 são destinados a pacientes pediátricos. Em parceria com a Secretaria de Saúde, foi estabelecida uma meta para atender à demanda de cirurgia pediátrica na região. Todas as internações ocorrem através do sistema de referência e contrarreferência, considerando a gravidade e especialidade cirúrgica, junto à Central de Regulação de Ofertas de Serviços de Saúde. Devido à fragilidade de rede venosa dessa população, os cateteres intravenosos são fundamentais para a administração de medicamentos e preservação da rede venosa em pacientes pediátricos (7). No entanto, há poucos estudos sobre o uso de dispositivos intravenosos centrais e suas complicações em crianças submetidas à cirurgia.

Este estudo apresentou limitações referentes aos registros dos procedimentos de inserção do cateter. Na instituição, os cuidados e procedimentos são registrados no prontuário eletrônico. No entanto, não existe um formulário específico com campos destinados ao registro de inserções e retirada do PICC. As anotações são feitas em seções abertas nos registros de enfermagem, tanto pelo enfermeiro responsável pelo procedimento quanto pela equipe técnica que acompanhou a inserção do dispositivo. É importante destacar que, devido à falta de padronização nas anotações da inserção e retirada do dispositivo, a aferição de desfecho pode apresentar variabilidade durante a análise dos resultados. Essa variabilidade foi encontrada na Suécia, entre março e abril de 2022. Práticas de inserção e manutenção de dispositivos intravenosos, quando não estão bem documentadas, podem gerar imprecisão para coleta de informações relacionadas (8,9). Na Austrália, entre 220 pacientes adultos avaliados, apenas o acesso venoso periférico possuía registro com formulário específico, com datas de inserção, remoção e recolocação. Não havia espaço no formulário para o mesmo registro de informação para PICC e cateter venoso central (9). 

Apesar da limitação encontrada nos registros de inserção e retirada dos dispositivos neste estudo, foi possível identificar a dispensação do PICC para o paciente por meio da central de custo da instituição. Essa abordagem revelou-se eficaz devido à estrutura organizada da central de custo, que mantém registros detalhados sobre a saída de materiais destinados aos pacientes. Cabe ressaltar que foram encontradas mais complicações (casos) do que a ausência de complicações (controle). Provavelmente, o perfil do hospital em atender pacientes pediátricos graves pressupõe que estes tenham maior chance de complicações do seria observado em outra instituição. Para contornar essa limitação, realizaram-se regressões logísticas ajustadas, incluindo regressão logística exata, para lidar com pequenos tamanhos de amostra.

Nesta pesquisa, as motivações de retirada do PICC incluíram obstrução, retirada acidental, presença de hiperemia, infecção de corrente sanguínea associada à linha central, trombose venosa profunda e óbito. Embora seguro, complicações podem surgir durante seu uso em todas as faixas etárias. Pesquisas nacionais e internacionais demonstraram semelhanças nas complicações encontradas neste estudo (3,10,11). Essas complicações podem interromper o tratamento e interferir na evolução clínica, prolongar a hospitalização, comprometer a segurança e a qualidade de vida do paciente, aumentar os custos assistenciais e sobrecarregar a equipe de saúde, uma vez que demandam atenção contínua dos profissionais e podem gerar impacto no Sistema Único de Saúde (12).

Entre as crianças, as que se mantiveram internadas na unidade de cuidados intensivos tiveram maior chance de complicações. É relevante mencionar que houve maior frequência de cirurgias cardíacas, e a comorbidade mais frequente foi a cardiopatia. A complexidade das cirurgias realizadas e o tempo prolongado de internação aumentaram as chances de complicações. Ainda sobre a análise multivariada, as crianças em uso de hidratação contínua também apresentaram maior chance para desenvolver complicações. Esse dado despertou a atenção em relação à manutenção do PICC, visto que o uso de hidratação intravenosa não substitui os protocolos de solução de infusão de solução salina e manutenção do dispositivo (13).

A obstrução do PICC foi a complicação mais frequente neste estudo. Achados semelhantes ocorreram em estudos nacionais e internacionais. No município do Rio de Janeiro, em uma instituição pública especializada em oncologia infantil, a obstrução ocorreu em 64,4% dos casos (11). Em 2023, na Indonésia, 40,0% das crianças apresentaram complicações, e, entre as complicações, a oclusão teve maior frequência (14).

Ao estudar os fatores associados à obstrução, identificou-se neste estudo que crianças do sexo feminino, com síndrome de Down e em uso de nutrição parenteral total tiveram maior chance de obstrução do dispositivo. Na literatura, o sexo é abordado de forma mais ampla, sem associação direta ao fator de obstrução ou complicação (15,17). Não foram encontrados estudos que associem diretamente a síndrome de Down com o fator de obstrução em cateteres de inserção periférica ou central. Porém, identificaram-se estudos que relataram a dificuldade de inserção de acesso intravenosos em crianças menores, especialmente aquelas com doenças complexas e presença de cardiopatia (18). Entre as quatro crianças com síndrome de Down, três apresentavam cardiopatia, o que pode ter influenciado os desfechos observados. 

Na China, um terço dos pacientes cardíacos pediátricos apresentou obstrução no dispositivo (19). Tais fatores podem representar um viés de confusão neste estudo, especialmente na associação entre as cardiopatias e o aumento de complicações. Embora não se tenha encontrado estudos semelhantes a este, acredita-se que o tamanho pequeno da amostra pode ter influenciado esse resultado. Um aspecto relevante é que todas as crianças com síndrome de Down foram internadas na unidade de cuidados intensivos e receberam hidratação intravenosa, fatores que, nesta análise, demostraram maior chances de complicações. Também foi observado que duas dessas crianças fizeram uso de nutrição parenteral total. Estes dados indicaram que a nutrição parenteral total está associada à maior chance de obstrução.

Com relação à utilização de nutrição parenteral total e o fator de obstrução, em 2019, entre os eventos adversos relacionados à administração de medicamentos via parenteral, mais da metade dos pacientes da pesquisa apresentou obstrução do dispositivo. Não foi possível determinar com precisão a proporção exata das complicações diretamente associadas à nutrição parenteral total (20). Foi sugerido que a associação da nutrição parenteral total com outros medicamentos, como antibióticos, aumenta o risco de oclusão devido ao depósito progressivo de microcristais ao longo da parede do cateter (20,21). Neste estudo, as crianças em quase sua totalidade mantinham uso de antibiótico. 

Dados de 2019 indicavam que pacientes que recebiam nutrição parenteral total como fonte primária de nutrição apresentavam alto risco de complicações infecciosas e não infecciosas relacionadas ao cateter, entre elas a obstrução dos dispositivos centrais (22). No Canadá, em 2021, as complicações mais comuns em pacientes pediátricos que utilizavam nutrição parenteral total foram a infecção de corrente sanguínea associada à linha central e a obstrução (23). O número de infecção de corrente sanguínea associada à linha central foi muito inferior quando comparado à obstrução (23). Outros fatores podem ser associados à obstrução de dispositivos centrais, como a educação permanente da equipe de enfermagem, o conhecimento e as habilidades dos enfermeiros, a solução e o método de infusão de solução salina e as técnicas de inserção, que não foram avaliados nesta pesquisa (15,20).

Em pacientes pediátricos, o uso do dispositivo intravenoso seguro é essencial para o tratamento eficaz. O PICC tem sido amplamente utilizado, tornando-se uma alternativa segura para o acesso venoso nesse grupo no ambiente hospitalar (3,24).

No contexto hospitalar, é importante que os registros de cuidados e a inserção de dispositivos intravenosos sejam devidamente registrados em prontuário. Essas informações são essenciais para a segurança dos pacientes e para detectar falhas no cuidado e na manutenção do dispositivo (25). Manter registros estruturados e padronizados em prontuários eletrônicos aumentam a precisão e qualidade dos registros de cateteres centrais, como PICC (8,26). Essa prática deve ser adotada antes e após o procedimento, pois lacunas nos registros representam um desafio para garantir a segurança do paciente (8).

A padronização dos materiais para inserção de PICC é essencial para garantir a qualidade e a segurança do procedimento. Sua ausência pode levar à aquisição de materiais inferiores e à necessidade de treinamento adicional da equipe de enfermagem. Na instituição onde o estudo foi conduzido, a padronização não ocorreu devido às limitações no sistema de compras. 

Os cateteres disponíveis eram de silicone e sem válvulas. A inclusão de válvulas, independentemente da posição, pode prevenir refluxo sanguíneo ao desconectar o cateter e reduzir o risco de oclusão. Ressalta-se que outros cuidados associados à presença da válvula devem ser realizados, como a infusão de solução salina após a administração de medicamentos intermitentes e hemoderivados (27).

Durante a pesquisa, observou-se que as complicações identificadas neste estudo eram comuns em hospitais pediátricos de nações desenvolvidas e em países em desenvolvimento (28,29). Entre os fatores associados no estudo, a obstrução pode estar relacionada ao uso de nutrição parenteral total. Cabe ao enfermeiro a responsabilidade de garantir a manutenção, permeabilidade e adequada via de administração para pacientes em uso de nutrição parenteral total (30). Além disso, manter hidratação intravenosa contínua no PICC não substitui a padronização de protocolo de solução de infusão de solução salina, que não havia na instituição. 

Conclui-se que pacientes em cuidados intensivos têm maior chance de complicações devido à criticidade dos casos, ao tempo prolongado de internação e às comorbidades. Manter hidratação intravenosa também demonstrou maior chance para a complicação. A identificação dos fatores associados à complicação da inserção do PICC neste estudo pode auxiliar os serviços de saúde quanto à priorização da realização do procedimento ou ao monitoramento mais intensivo quando a inserção é necessária. Tais ações poderão beneficiar os pacientes e melhorar o desempenho do Sistema Único de Saúde.

## Data Availability

O dado não está disponível, pois o acesso é restrito ao hospital.
